# Intradiploic Epidermoid Cyst Causing Transverse Sinus Compression in an Adolescent With Papilledema: A Rare Mimic of Idiopathic Intracranial Hypertension

**DOI:** 10.7759/cureus.97920

**Published:** 2025-11-27

**Authors:** Jedediah Bondy, Sammy Khalouf, Mansi Shah, Paul Dobria, Eric Spitzer

**Affiliations:** 1 Radiology, Lake Erie College of Osteopathic Medicine, Erie, USA; 2 Urology, Lake Erie College of Osteopathic Medicine, Erie, USA; 3 Diagnostic Radiology, Sidney Kimmel Medical College, Philadelphia, USA; 4 Radiology, Rochester Regional Health, Rochester, USA

**Keywords:** intradiploic epidermoid cyst, papilledema, pediatric neuroimaging, secondary intracranial hypertension, venous sinus compression

## Abstract

Idiopathic intracranial hypertension (IIH) typically presents in young, obese women with symptoms such as headache, pulsatile tinnitus, and visual disturbances due to raised intracranial pressure. While IIH is commonly diagnosed in the absence of identifiable pathology, it is essential to rule out secondary causes that may mimic its presentation. Because calvarial lesions causing venous outflow obstruction are rare but clinically significant, early imaging is critical to differentiate secondary from idiopathic cases.

We report the case of a 16-year-old female with a history of obesity, hypertension, and prediabetes who presented with a two-month history of daily headaches associated with ocular pain, photophobia, and intermittent blurred vision. Ophthalmologic examination revealed bilateral grade III papilledema. MRI of the brain identified an intradiploic epidermoid inclusion cyst within the right occipital bone, causing compression of the dominant right transverse sinus, with a diminutive left transverse sinus further compromising venous outflow. These findings were consistent with secondary intracranial hypertension due to extrinsic venous sinus compression. The diagnostic progression from CT to MRI clarified that a structural lesion, not intrinsic stenosis, explained the patient’s presentation. The patient was started on acetazolamide, with additional evaluation planned, including MR venography (MRV) and lumbar puncture.

This case highlights the importance of considering structural causes in patients who present with IIH-like symptoms. Venous sinus stenosis, whether intrinsic or extrinsic, can impair cerebrospinal fluid (CSF) absorption and elevate intracranial pressure. Intradiploic epidermoid cysts are rare, particularly in pediatric patients, but may subtly obstruct venous drainage when located adjacent to major dural sinuses. Comparison with prior reports underscores that even small calvarial lesions can produce clinically significant outflow obstruction.

This case underscores the need for thorough neuroimaging, including MRV, in patients with suspected IIH to exclude secondary causes. Strengthening the diagnostic link between symptoms, imaging, and venous sinus pathology ensures prompt management and reduces the risk of permanent visual morbidity.

## Introduction

Idiopathic intracranial hypertension (IIH), or pseudotumor cerebri, is defined as elevated intracranial pressure (ICP) in the absence of an identifiable cause such as hydrocephalus, hypertensive encephalopathy, intracranial mass, or infection [1]. IIH most commonly affects young obese females and typically presents with headaches, papilledema, pulsatile tinnitus, and transient vision changes [1,2]. These hallmark features serve as the foundation for distinguishing IIH from secondary causes of intracranial hypertension.

Although the pathophysiology of IIH is not fully understood, multiple studies support an association between IIH and transverse sinus stenosis, leading to impaired venous outflow and decreased CSF absorption via arachnoid granulations [3,4]. In most cases, this narrowing is intrinsic or functional. However, rare instances of extrinsic sinus compression by structural lesions have been reported and may mimic the clinical features of IIH [5]. Although uncommon, calvarial lesions adjacent to major venous sinuses, including epidermoid cysts, have been documented as causes of secondary intracranial hypertension.

Epidermoid inclusion cysts are benign, slow-growing lesions derived from ectodermal remnants that arise within the diploic space of the calvaria. As they expand, they may erode surrounding bone and exert mass effect on intracranial structures. When located near critical venous drainage pathways such as the transverse sinus, they may obstruct venous outflow and lead to secondary intracranial hypertension [6,7].

We present the case of a 16-year-old female with classic IIH symptomatology who was found to have an intradiploic epidermoid cyst compressing the dominant right transverse sinus, resulting in bilateral papilledema and symptoms of raised ICP. This case highlights the importance of maintaining a broad differential diagnosis even in patients who match the typical demographic profile for IIH. To our knowledge, this is one of the few reported cases of secondary intracranial hypertension due to a calvarial inclusion cyst in the pediatric population.

## Case presentation

A 16-year-old female with a history of obesity (BMI >99th percentile), hypertension, prediabetes, and iron deficiency anemia presented with a two-month history of daily frontal headaches associated with eye pain, photophobia, pulsatile tinnitus, and intermittent blurry vision. She denied diplopia, visual obscurations, floaters, or ocular trauma. Her symptoms were initially managed conservatively as migraine, but progression prompted referral for further evaluation.

Fundoscopic examination demonstrated grade III papilledema with obscuration of the superior and inferior vessels bilaterally. Visual acuity was 20/20 bilaterally, and no relative afferent pupillary defect was present. On arrival, her blood pressure was 166/92 mmHg, and her heart rate was 102 bpm. Neurological examination was non-focal.

**Figure 1:**
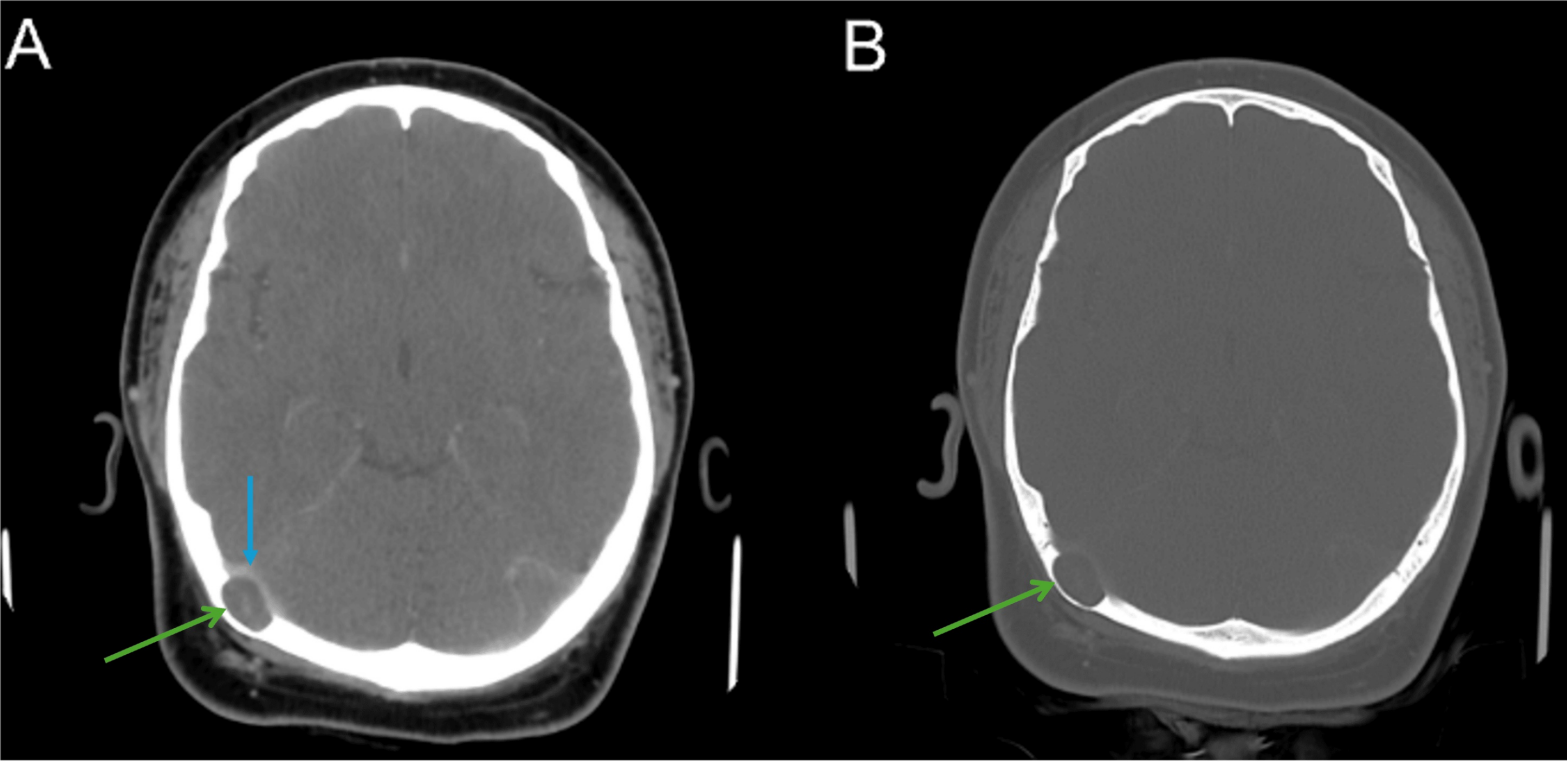
Soft-tissue (A) and bone (B) algorithm axial contrast-enhanced CT images of the head demonstrate a 1.8 cm intradiploic lucent lesion (green arrows) within the right occipital calvarium. The lesion markedly thins the inner table (B) and exerts mass effect on the right transverse sinus (blue arrow), suggesting extrinsic venous compression.

**Figure 2:**
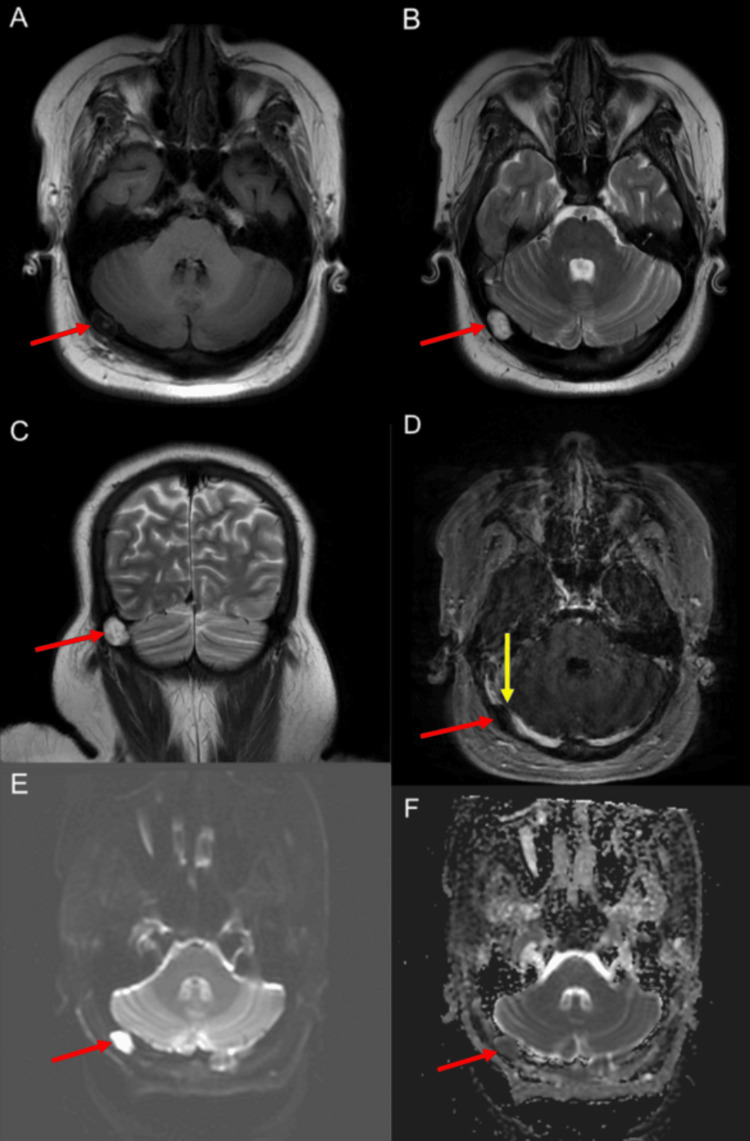
Contrast-enhanced MRI of the brain shows a 1.6 × 1.0 cm lesion within the right parietal/occipital calvarium that is low on T1 and high on T2 (A-C), non-enhancing (D), and diffusion-restricting (E-F). The lesion thins both the inner and outer tables (B, C) and narrows the right transverse sinus (yellow arrow). Imaging characteristics are consistent with an intradiploic epidermoid inclusion cyst (red arrows).

Non-contrast CT of the head showed a 1.8 cm lucent lesion in the right occipital bone with thinning of the inner table and apparent herniation of parenchyma into the calvarium (Figure 1). MRI was later obtained and revealed a 1.6 × 1.0 cm T2 hyperintense, non-enhancing, diffusion-restricting intradiploic lesion consistent with an epidermoid cyst (Figure 2). The mass caused extrinsic compression of the dominant right transverse sinus; the left transverse sinus was diminutive.

These combined findings established secondary intracranial hypertension due to venous sinus compression. The patient was started on acetazolamide 500 mg twice daily. MR venography (MRV) and lumbar puncture were planned to quantify ICP elevation and further guide management. Follow-up was arranged with neurology, ophthalmology, and nutrition services.

## Discussion

This case illustrates a rare structural cause of secondary intracranial hypertension in a pediatric patient whose presentation closely mimicked IIH. Her age, obesity, and symptoms - headache, papilledema, and pulsatile tinnitus - were typical of IIH, yet neuroimaging revealed an intradiploic epidermoid cyst compressing the dominant right transverse sinus.

The transverse sinuses are critical for cerebral venous outflow, and compromise - whether intrinsic narrowing or extrinsic compression - can elevate venous pressure and impair CSF absorption [3,4]. Although intrinsic stenosis is frequently seen in IIH, structural causes must be excluded before assigning the diagnosis [5].

Epidermoid inclusion cysts arising in the diploic space may erode bone and compress adjacent venous sinuses. When this occurs near a dominant drainage pathway, even a small lesion may produce significant ICP elevation [6,7]. In this patient, a diminutive left transverse sinus amplified the hemodynamic impact of right-sided compression, resulting in bilateral papilledema.

Pediatric cases of intradiploic epidermoid cysts causing IIH-like symptoms are exceedingly rare, yet failing to identify a secondary cause may lead to delayed treatment and permanent visual morbidity. As demonstrated here, MR imaging with MRV is essential in evaluating papilledema, even in patients who appear to fit the classic demographic profile for IIH.

Management of secondary intracranial hypertension differs from idiopathic disease. While acetazolamide may offer temporary relief, definitive treatment often requires surgical removal of the compressive lesion, and venous stenting may be considered in select cases [8]. MRV and lumbar puncture were planned to further characterize venous flow and ICP elevation, helping guide long-term management.

## Conclusions

This case highlights the need for a broad differential diagnosis in patients with papilledema and elevated ICP, even when they match the typical demographic profile for IIH. In this pediatric patient, an intradiploic epidermoid cyst compressing the dominant transverse sinus produced a clinical picture nearly identical to IIH, leading to a diagnosis of secondary intracranial hypertension.

Structural venous outflow obstruction can mimic IIH but requires different management, underscoring the importance of early neuro-imaging with MRI and MRV. Prompt identification of secondary causes is essential to prevent irreversible visual loss and guide appropriate treatment.
